# Human umbilical cord mesenchymal stem cells attenuate liver fibrosis in mice and inhibit hepatic stellate cell activation by secreting soluble factors

**DOI:** 10.1186/s13287-025-04812-6

**Published:** 2025-11-27

**Authors:** Qi Zhou, Junyu Wang, Ke Cheng, Ruyi Mei, Janette Heegsma, Han Moshage, Martin C. Harmsen, Klaas Nico Faber, Pingnan Sun

**Affiliations:** 1 University Medical Center Groningen, Department of Gastroenterology and Hepatology, University of Groningen, Groningen, The Netherlands; 2 University Medical Center Groningen, Department of Pathology and Medical Biology, University of Groningen, Groningen, The Netherlands; 3 Department of Gynecology of the First Affiliated Hospital, Shantou University Medical College, Shantou, Guangdong China; 4 Department of Stem Cell Research Center, Shantou University Medical College, Shantou, Guangdong China; 5 W.J. Kolff Institute for Biomedical Engineering and Materials Science, University of Groningen, University Medical Centre Groningen, Groningen, The Netherlands; 6 University of Groningen, University Medical Centre Groningen, Groningen Research Institute for Asthma and COPD (GRIAC), Groningen, The Netherlands

**Keywords:** Mesenchymal stem cells, Liver fibrosis, Hepatic stellate cells, Secreted soluble factors, Cell-free therapy

## Abstract

**Background:**

Mesenchymal stem cells (MSCs) have emerged as promising candidates to treat clinical liver fibrosis. However, the key factors and mechanisms underlying their antifibrotic effects remain largely unclear. Our aims were to assess the therapeutic efficacy of human umbilical cord (UC) MSCs in a murine liver fibrosis model, and to dissect the influence of their secretome in vitro.

**Methods:**

Liver fibrosis was induced in mice using carbon tetrachloride (CCl₄). Fluorescent labeling was used to track UC-MSC distribution post-injection, comparing intraperitoneal (IP) and intravenous (IV) delivery. UC-MSCs were administered intraperitoneally weekly, starting at either fibrosis induction (prevention) or after six weeks (therapeutic). Fibrosis severity was evaluated by hematoxylin and eosin (H&E) staining, Masson’s trichrome staining and immunohistochemical (IHC) staining for alpha smooth muscle actin (α-SMA). Human hepatic stellate LX-2 cells were treated with complete UC-MSC-conditioned medium (CM^UC−MSC^), its soluble factors (SF^UC−MSC^) or extracellular vesicle fraction of the secretome (EV^UC−MSC^). Fibrosis-related gene and protein expression were analyzed by Q-PCR, immunofluorescence, and Western blotting.

**Results:**

IP-injected UC-MSCs showed increased liver-specific accumulation compared to IV-injected UC-MSC that distributed mostly to the lungs. In vivo, IP-injected UC-MSC significantly reduced fibrosis, as evidenced by decreased histological damage, collagen deposition, and α-SMA expression. Both preventive and therapeutic UC-MSC IP-treatments were effective. In vitro, CM^UC−MSC^ suppressed TGF-β-induced *ACTA2*/α-SMA expression and proliferation of human LX-2 cells, but not *COL1A1*/collagen type I expression. These effects persisted in EV-depleted SF^UC−MSC^. EV^UC−MSC^ inhibited proliferation, but did not suppress *ACTA2*/α-SMA or *COL1A1*/collagen type I levels in LX-2 cells.

**Conclusions:**

IP-administered therapeutic UC-MSCs preferentially accumulate in the liver and effectively prevent and reverse liver fibrosis in mice, while inhibiting hepatic stellate cell activation in vitro. The antifibrotic effects in vivo are likely primarily mediated by excreted soluble factors rather than extracellular vesicles.

**Supplementary Information:**

The online version contains supplementary material available at 10.1186/s13287-025-04812-6.

## Introduction

Liver fibrosis is a pathological consequence of chronic liver injury, characterized by excessive deposition of extracellular matrix (ECM) and disruption of liver architecture [1]. It is also a significant health burden worldwide as it may progress to cirrhosis, liver failure and hepatocellular carcinoma. Despite advances in understanding the mechanisms underlying liver fibrosis, effective therapeutic options remain limited. Mesenchymal stem cells (MSCs) have emerged as a promising therapeutic candidate due to their regenerative and immunomodulatory properties [2, 3]. Preclinical and clinical studies have demonstrated the efficacy of MSCs in treating liver diseases, including cirrhosis and liver failure [3–5].

Among the various sources of MSCs, those derived from the human umbilical cord (UC-MSCs) have garnered considerable attention due to their potent therapeutic potential and ease of isolation. UC-MSCs adhere to the criteria established by the International Society for Cellular Therapy (ISCT), exhibiting spindle-shaped morphology, specific surface marker expression (positive for CD73, CD90, and CD105, and negative for CD34, CD19, CD45, CD11b, and HLA-DR), and the ability to differentiate into osteocytes, adipocytes, and chondrocytes [6]. These cells also secrete a diverse array of bioactive molecules and extracellular vesicles (EVs) that modulate cellular communication and tissue repair [7, 8].

Although MSCs have demonstrated promising therapeutic effects in liver fibrosis already [9], further research is needed to understand their distribution and homing mechanisms in vivo. Moreover, the therapeutic efficacy of MSCs fundamentally depends on their capacity to either physically localize to sites of injury or exert functional effects on damaged tissues [10]. In preclinical mouse models of liver fibrosis, MSC are typically introduced IV via tail vein injections, while induction of liver fibrosis is induced be IP-injected CCl_4_ [11, 12]. IV-injected MSC show strong accumulation in lungs, potentially limiting the therapeutic effect of MSCs. Therefore, it is essential to investigate and compare the distribution and homing of MSCs following different routes of administration, in particular via intravenous and intraperitoneal injections.

The effects of MSCs on liver fibrosis encompass both their direct differentiation into hepatocytes and their regulatory influence on immune cells, hepatic stellate cells (HSCs), and other related cells through cellular or paracrine mechanisms [13, 14]. The predominant therapeutic impact of MSCs is mediated by their paracrine actions rather than direct differentiation [15]. A growing body of evidence highlights the therapeutic potential of the MSC secretome [16]. The secretome mediates many of the therapeutic effects of MSCs, such as anti-apoptosis, anti-inflammation, and anti-senescence, allowing the development of cell-free strategies [17]. In liver fibrosis, the MSC secretome has been shown to inhibit hepatic stellate cell (HSC) activation, a key driver of fibrogenesis [18]. Activated HSCs are characterized by increased expression of α-smooth muscle actin (α-SMA) and ECM proteins, such as collagen type I, contributing to the progression of liver fibrosis [19]. However, the specific functional components of the secretome and the relative contributions of soluble factors versus EVs in mediating these anti-fibrotic effects remain unclear, representing a critical knowledge gap in the field [20].

In this study, we aimed to investigate the therapeutic potential of UC-MSCs and their secretome in a carbon tetrachloride (CCl₄)-induced liver fibrosis model. We evaluated the effects of UC-MSC-derived conditioned medium (CM^UC−MSC^), soluble factors (SF^UC−MSC^), and extracellular vesicles (EV^UC−MSC^) on HSC activation and proliferation using the human LX-2 stellate cell line. These findings will provide new insights into the mechanisms of MSC-mediated anti-fibrotic effects and highlight the potential of cell-free therapies for liver fibrosis.

## Materials and methods

The work has been reported in line with the ARRIVE guidelines 2.0.

### Isolation and culture of MSCs

MSCs were obtained from fresh UC tissue of healthy donors. The UC tissue was collected under ethical approval and patient consent. All procedures were approved by the Ethics Committee of the Second Affiliated Hospital of Shantou University Medical College (Ethics Approval No.2021-89). In vitro MSCs were isolated from the umbilical cords from the UMCG EC facility. The extraction of MSCs was based on the description reported previously [18]. In brief, UC segments (5–10 cm) were sectioned longitudinally to expose Wharton’s jelly. The tissue was incised with a sterile scalpel to enhance contact with the culture surface and placed in a 10 cm² Petri dish. The tissues were cultured in Dulbecco’s modified Eagle’s medium (DMEM; Thermo Fisher Scientific, Waltham, Massachusetts, USA) supplemented with 10% fetal bovine serum (FBS; Thermo Fisher Scientific), 2 mM L-glutamine (Thermo Fisher Scientific), and 0.5% antibiotic–antimycotic solution (Thermo Fisher Scientific) at 37 °C in a 5% CO₂ humidified atmosphere. After 5 days, the tissues were removed, and the medium was replaced. Cells were expanded until 80–90% confluence, harvested using TrypLE Select solution (Thermo Fisher Scientific), and characterized for phenotype and differentiation potential. Cells were passaged up to 10 times for experiments.

### MSC tri-lineage differentiation

MSCs were assessed for adipogenic, osteogenic, and chondrogenic differentiation using a human MSC differentiation kit (TBDscience, Tianjin, China). The protocol was performed as described previously [21]. Briefly, Adipogenic differentiation was induced at 100% confluence and confirmed by Oil Red O staining after 3–4 weeks (Cat# TBD20190004). Osteogenic differentiation was induced at 60–70% confluence and confirmed by Alizarin Red staining after 3–4 weeks (Cat# TBD20190002). Chondrogenic differentiation was performed in pellet cultures and confirmed by Alcian Blue staining after 3–4 weeks (Cat# TBD20190003).

### MSC labelling with DIR dye

Phenotypic analysis: Cells were washed with PBS, dissociated with TrypLE (Thermo Fisher Scientific), and collected by centrifugation at 200 x g for 2 min. Cells were stained with FITC- or PE-conjugated antibodies against CD73, CD90, CD105, CD34, CD31, CD14, CD45, and HLA-DR (4 A Biotech, Beijing, China). Cells were fixed in 4% paraformaldehyde, incubated with antibodies for 30 min at 4 °C, and analyzed using a BD C6 flow cytometer (BD Pharmingen, Franklin Lakes, NJ, USA). Data were processed using FlowJo VX software (Tree Star, Ashland, OR, USA). Data were analyzed using the FlowJo VX program (Tree Star).

MSC labelling with dir dye: DiR (1,1′-Dioctadecyl-3,3,3′,3′-tetramethylindotricarbocyanine iodide) staining stock solution was used to label cell membranes of MSCs following the manufacturer instructions (DiR dye kit, eBioscience, San Diego, CA, USA) by adding 180 µL of dimethyl sulfoxide (DMSO, Sigma-Aldrich, St. Louis, MO, USA) to the DiR dye to make a 5 mM stock. MSCs were labeled with DiR dye (eBioscience) by incubating 1 µL of DiR stock solution (5 mM in DMSO) with cells in PBS for 15 min. Unbound dye was removed by washing with PBS and centrifugation at 200 × g. The fluorescence of MSCs were examined under a fluorescent microscope (Leica Microsystems, Wild Heerbrugg, Germany). The labeled MSCs were detected in mice using the IVIS Living Image System (PerkinElmer, USA) and analyzed with Living Image software.

### CCl_4_-induced mouse model of liver fibrosis and treatment of MSCs

#### Experimental design and groups

As a potent liver fibrosis-inducing hepatotoxin, CCl_4_ has been used to generate chemical-induced liver fibrosis in mice [22]. Inbred male C57Bl/6 mice were housed in the animal facility of Shantou University. All procedures were approved by the Ethics Committee of Shantou University Medical College (SUMCXM2024-207). To induce liver fibrosis (CCl_4_ group), mice (four to six weeks old) were injected intraperitoneally with 0.6 ml CCl_4_ per kg (Sigma-Aldrich) in olive oil (3 ml olive oil per 1 ml CCl_4_), twice per week for 6 or 10 weeks. Untreated mice were used as controls. The oil group administered olive oil at 0.6 ml per kg.

The mice were tested in two batches. For the first batch (prevention), 2 million MSCs were resuspended in 100 µL PBS and injected intraperitoneally once a week (24 h after CCl_4_ injection). For the second batch (therapeutic), MSC treatment was initiated after 12 CCl_4_ injections (24 h after CCl_4_ injection), and continued once a week (24 h after CCl_4_ injection). For the first batch, mice were sacrificed, and samples were harvested after 6 and 10 weeks. For the second batch, mice were sacrificed, and samples were harvested after 10 weeks.

All mice were anesthetized with isoflurane and subsequently euthanized by cervical dislocation in accordance with institutional ethical guidelines. A portion of the liver was snap frozen in liquid nitrogen and stored at -80 ℃ until RNA and protein were isolated. Another portion was fixed in 4% paraformaldehyde, embedded in paraffin, and sectioned for further analysis according to standard procedures.

### H&E staining and Masson staining

Liver tissues were fixed in 4% paraformaldehyde, paraffin-embedded, and sectioned (4 μm). Sections were stained with hematoxylin and eosin (H&E) or Masson’s trichrome (Solarbio, Beijing, China) according to the suppliers’ protocols and imaged using a light microscope. Collagen deposition was quantified using ImageJ software.

### Immunohistochemistry (IHC) staining

Sections were stained with an α-SMA antibody (see Table S2) and counterstained with hematoxylin. IHC-stained α-SMA was quantified by using ImageJ image analysis software.

### Preparation of conditioned medium (CM^UC-MSC^), extracellular vesicle (EV^UC-MSC^) and soluble factor (SF^UC-MSC^) isolation from UC-MSC

MSCs were cultured in serum-free medium for 48 h after to 80% confluence to obtain CM^UC−MSC^. The CM^UC−MSC^ was subjected to differential ultracentrifugation (Beckman, Indianapolis, IN, USA) to obtain EVs, as previously described [23]. Briefly, samples were thawed at room temperature (24 °C), transferred into 38 mL (13 × 51 mm) tubes (#344057, Beckman Coulter Inc., Brea, CA, USA), and centrifuged for 30 min at 20,000 ×g. The resulting supernatants were carefully collected as SF^UC−MSC^ for experiments and centrifuged at 110,000 ×g at 4 ℃ for 2 h. Pellets from this centrifugation step were washed in PBS, pooled and centrifuged again for 1 h at 110,000 ×*g* at 4 ℃. The final pellet was resuspended in 100 µL of PBS (EV^UC−MSC^) and stored in aliquots at -80 ℃ or directly into lysis buffer for analysis. Protein concentration in the EV pellet was measured using a microBCA protein assay kit (Thermo Scientific). Before treatment, CM^UC−MSC^ and SF^UC−MSC^ was filtered through 0.2 μm sterile filters (Whatman, Maidstone, UK). LX2 serum-free medium served as a control.

### Nanoparticle tracking analysis (NTA)

The concentration and size distribution of isolated EV^UC−MSC^ were assessed using nanoparticle tracking analysis (NTA) with NanoSight NS300 instrumentation (Malvern Panalytical, Malvern, UK). NTA procedure was performed as described previously [23]. EV samples were diluted with PBS to achieve an estimated concentration ranging from 10^8^ to 10^9^ particles/mL in a total volume of 1 mL. Each sample underwent continuous flow through a flow-cell top plate at 18 ℃ using a syringe pump. At least five 10-second videos documenting the Brownian motion of nanoparticles were recorded, and a minimum of 1,000 completed tracks were analyzed using NanoSight software (NTA v3.2).

### Cryogenic electron microscopy

The morphology and structural integrity of EVs were assessed using cryogenic electron microscopy (Cryo-EM) as described in previous studies [24]. EV samples were prepared by diluting them to a suitable concentration (10^11^ particles/mL) with PBS. A few micro liters sample was placed on a holy carbon coated grid (Quantifoil 3.5/1) and vitrified in ethane using a Vitrobot. Samples were observed in a Tecnai T20 electron microscope operating at 200 keV. Images were recorded under low-dose conditions with a slow scan CCD camera. Morphological features, including EV size and membrane integrity, were analyzed.

### Treatment of activated human LX-2 cells

The LX-2 cell line (SCC064, Merck, Amsterdam, the Netherlands), a widely used human hepatic stellate cell (HSC) described in studies by Xu et al. and Smith-Cortinez et al. [25, 26] was utilized between passages 22 and 32 for the experiments. The cells were maintained in high-glucose Dulbecco’s Modified Eagle Medium (DMEM, Thermo Fisher) supplemented with 10% fetal bovine serum (FBS, Thermo Fisher) and 1× Penicillin–Streptomycin (Thermo Fisher), which served as the standard culture medium [27]. LX-2 cells were cultured to 80% confluence and treated with 5 ng/ml transforming growth factor beta (TGF-β, R&D Systems, Minneapolis, MN, USA) alone or in combination with CM^UC−MSC^, SF^UC−MSC^, or EV^UC−MSC^ for 48 h. Cells were harvested for RNA, protein, and immunofluorescence microscopy analysis.

### Cell proliferation measurement

Proliferation of LX-2 cells was assessed by a Real-Time xCELLigence system (RTCA DP; ACEA Biosciences Inc., Santa Clara, CA, USA). The procedure was performed as described previously [23]. Cells were seeded in a 16-well E-plate and treated as indicated. The cell index, reflecting changes in impedance, was monitored continuously using the xCELLigence system. Proliferation was analyzed using RTCA Software Pro (ACEA Biosciences Inc.) with baseline normalization. The Relative Normalized Cell Index (RNCI) was derived from the slope of linear growth phases for inter-condition comparisons.

### RNA isolation and quantitative real-time reverse transcription polymerase chain reaction (qRT-PCR)

Total RNA extraction and reverse transcription to cDNA were performed following previously established protocols [28]. Total RNA was extracted from cells using TRI Reagent (Sigma-Aldrich) after washing with PBS. RNA concentration was measured using a NanoDrop 2000c spectrophotometer (Thermo Fisher Scientific). For cDNA synthesis, 2.5 µg of total RNA was reverse transcribed in a 50 µL reaction volume containing 1x RT buffer, 1 mM dNTPs, 10 ng/µL random nanomers, 0.6 U/µL RNaseOUT, and 4 U/µL M-MLV reverse transcriptase (all from Thermo Fisher Scientific). The resulting cDNA was diluted 20-fold in nuclease-free water, and qPCR was performed on a StepOnePlus system (Applied Biosystems) using TaqMan probes and primers (sequences in Table S1) [27]. Each reaction contained 2× reaction buffers (Eurogentec), 5 µM probe, and 50 µM primers. mRNA levels were normalized to 18 S rRNA.

### Western blot analyses

To assess the expression of specific proteins, Western blotting experiments were performed on cell lysates as described by Natalia et al. [25]. Cell lysates were resolved on Mini-PROTEAN TGX Stain-Free Precast Gels (Bio-Rad, Veenendaal, The Netherlands) and transferred to nitrocellulose membranes using a Trans-Blot Turbo System (Amersham Biosciences, Piscataway, NJ, USA). Protein transfer was verified by Ponceau S staining (0.1% w/v) (Sigma-Aldrich). Membranes were probed with primary antibodies (See Table S2, dilution of 1:1000), followed by peroxidase-conjugated secondary antibodies (See Table S2, dilution of 1:2000). α-Tubulin or GAPDH was used as the loading control. Protein bands were detected using a ChemiDoc XRS system (Bio-Rad) and quantified with ImageJ software.

### Immunofluorescence microscopy

Immunofluorescence analyses were performed as described previously [25]. LX-2 cells were cultured on glass coverslips in 12-well plates. After treatment, cells were washed twice with PBS and fixed in 4% paraformaldehyde for 10 min at room temperature. Following PBS washes, cells were permeabilized with 0.1% Triton X-100/PBS for 30 min at 37 °C. Non-specific binding was blocked with 1% BSA/PBS for 30 min. Primary antibodies (see Table S2, dilution of 1:200) were applied for 1 h at room temperature. After washing, cells were incubated with secondary antibodies (see Table S2, dilution of 1:400) for 30 min in the dark. Coverslips were mounted with DAPI-containing ProLong antifade reagent (Vector Laboratories, Inc., Peterborough, UK) and imaged by fluorescence microscopy (Leica Microsystems, Wild Heerbrugg, Germany) using Leica ALS AF software (Leica, Amsterdam, The Netherlands).

### Statistical analysis

Analyses were performed using GraphPad Prism 9 (version 9.0.0, GraphPad Software, Inc., USA) and IBM SPSS Statistics (version 26, IBM Corp., USA). Results presented represent the mean of at least three independent experiments and are expressed as the mean ± standard error of the mean (SEM) for continuous variables. Statistical significance was assessed using either Student’s t-test or one-way analysis of variance (ANOVA), followed by Fisher’s least significant difference (LSD) test for post-hoc analysis. A significance threshold of *p* < 0.05 was applied to determine statistical significance.

## Results

### MSCs accumulate in the liver following intraperitoneal injection

**Fig. 1:**
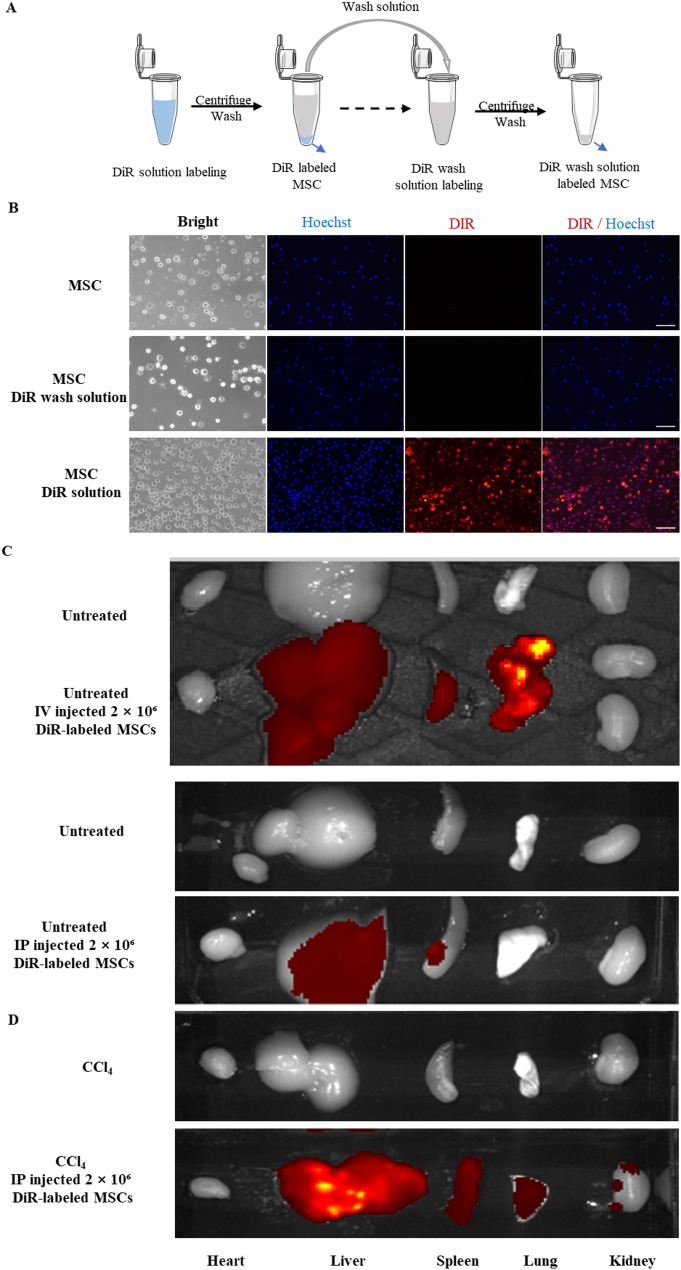
MSCs were enriched in liver after IP injection. MSC-related signals were detected by live imaging 7 days after intraperitoneal injection of MSC. **A** Schematic diagram for MSC labeling with DiR and DiR wash solution. **B** Representative cell images of MSCs stained with DiR or DiR wash solution. Unstained MSCs served as controls. Scale bar: 100 μm. **C** Detection of fluorescence signals by live imaging after tail vein (IV) or intraperitoneal (IP) injection in untreated mice. **D** Detection of fluorescence signals by live imaging after intraperitoneal (IP) injection in CCl_4_ treated mice. Fluorescence signals as detected by live imaging 7 days after cell administration

Human UC-MSCs were isolated and their multipotent differentiation capabilities and phenotype were characterized (Fig. S1). To confirm that the fluorescent signal specifically labeled MSCs, we collected the DiR wash solution (supernatant after washing and centrifugation) and stained MSCs, while DiR-stained MSCs served as positive control and untreated cells served as a negative control (Fig. 1A). Only DiR-labeled MSCs exhibited fluorescence while DiR- wash solution labeled MSC, and controls did not. (Fig. 1A, B). Consistently, in vivo fluorescence imaging following MSC injection into mice demonstrated a similar fluorescence pattern, further validating the specificity of the labeling (Fig. S2). To determine the optimal method for hepatic engraftment of MSCs, we compared two methods: tail vein (IV) injection and intraperitoneal (IP) injection. The tracing results of fluorescently labeled MSCs showed that MSCs were mainly distributed in the lungs after IV injection via the tail vein. In contrast, MSCs engrafted preferentially in the liver after i.p. injection, with low levels in lung and other organs (Fig. 1C). Thus, the i.p. injection method was used in all following experiments. Seven (7) days after i.p. administration, UC-MSCs had accumulated in the spleen, lungs, and kidneys but predominantly in the liver (Fig. 1C&D). Notably, MSCs appeared to accumulate in greater numbers in fibrotic livers compared to normal livers (Fig. 1D), suggesting a potential tropism toward fibrotic tissue.

### MSCs prevent CCl_4_-induced liver fibrosis in vivo

**Fig. 2:**
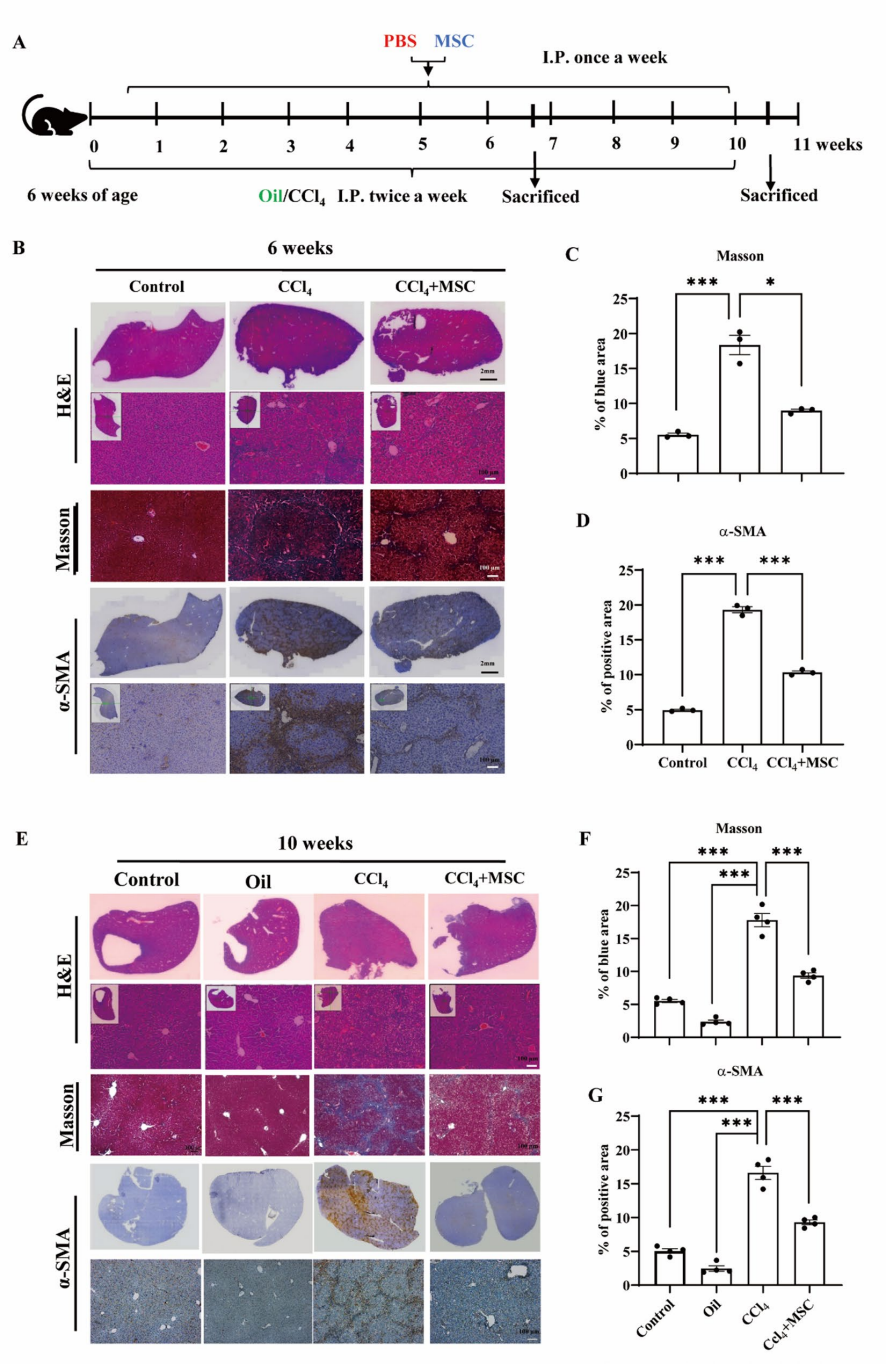
MSC administration prevents CCl_4_-induced liver fibrosis in vivo. **A** Schematic diagram of experimental procedures and time points. **B** Representative images of Masson staining, IHC staining (a-SMA), and hematoxylin and eosin-stained liver sections of the control group, CCl_4_ group, and CCl_4_ + MSC group after 6 weeks of treatment. **C**, **D** ImageJ was used for quantitative analysis of Masson and a-SMA IHC-stained positive areas. Results are the mean ± SEM (*n* = 4). **E** Representative images of Masson staining, IHC staining (a-SMA), and hematoxylin and eosin-stained liver sections of the control group, oil group, CCl_4_ group, and CCl_4_ + MSC group after 10 weeks of treatment. **F**, **G** ImageJ was used for quantitative analysis of Masson and a-SMA IHC-stained positive areas. Results are the mean ± SEM (*n* = 4). One-way ANOVA was used to compare differences among three or more groups (*P < 0.05, **P < 0.01, ***P < 0.001)

Mice injected with CCl_4_ showed increased liver scarring, enhanced myofibroblast activation (evidenced by elevated *ACTA2* and *COL1A1* gene expression, and elevated α-SMA protein levels), and elevated serum liver enzymes (alanine aminotransferase (ALT) and aspartate aminotransferase (AST)), compared to the control group (Fig. S3). To measure the preventive effect of MSCs, we injected MSCs one day after the last CCl_4_ injection each week and sacrificed the mice after 6 and 10 weeks (Fig. 2A). Compared with the CCl_4_ group, mice injected with MSCs exhibited decreased liver scarring, reduced myofibroblast activation (indicated by α-SMA IHC), and lower levels of collagen deposition (indicated by Masson staining) after 6 weeks (Fig. 2B–D). The same phenomenon was observed after 10 weeks (Fig. 2E–G).

### MSCs attenuate CCl_4_-induced liver fibrosis in vivo

**Fig. 3:**
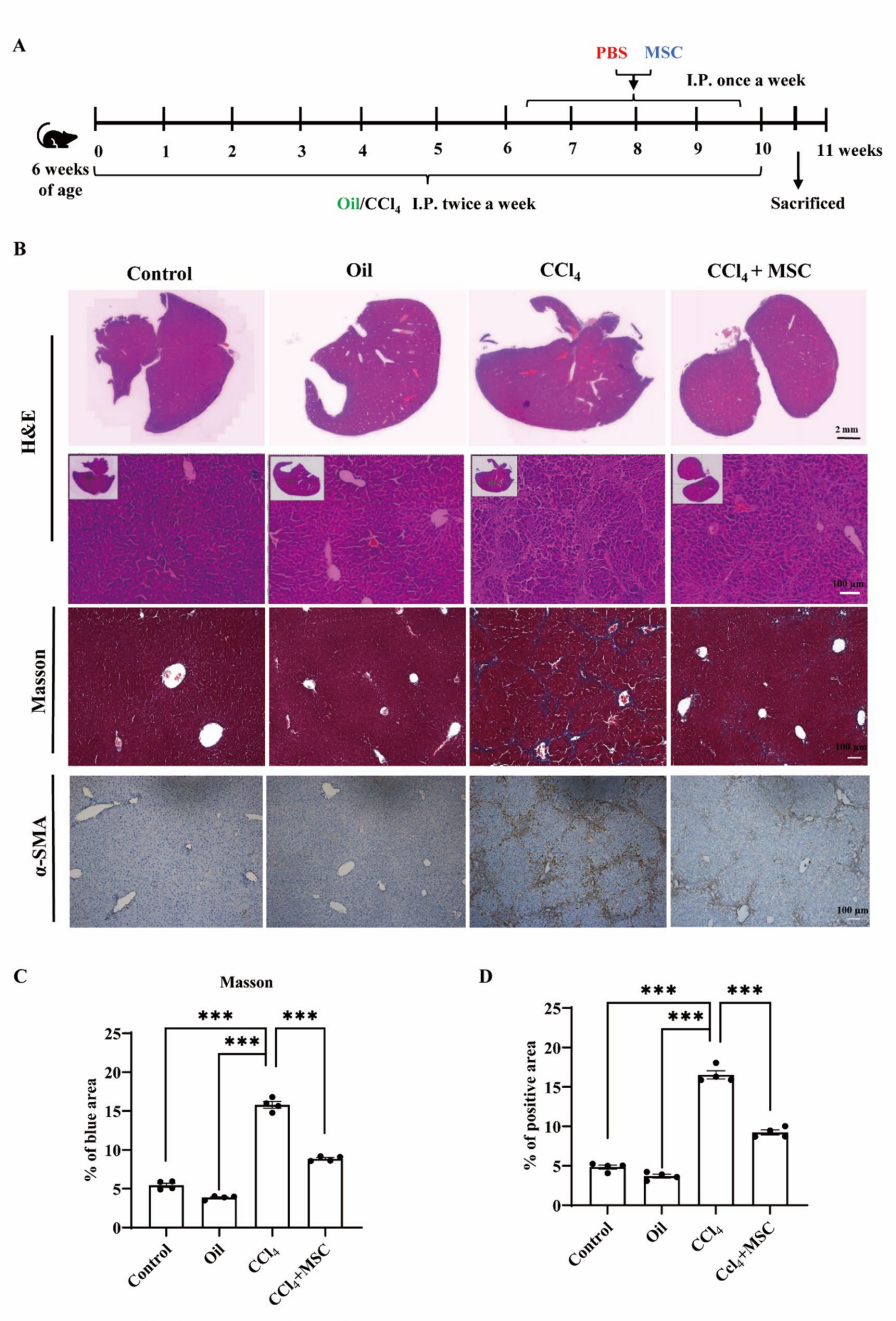
MSCs attenuate CCL_4_-induced liver fibrosis in vivo. **A** Schematic diagram of experimental procedures and time points. **B** Representative images of Masson staining, IHC staining (a-SMA), and hematoxylin and eosin-stained liver sections of the control group, oil group, CCl_4_ group, and CCl_4_ + MSC group after 10 weeks of treatment. **C**, **D** ImageJ was used for quantitative analysis of Masson and a-SMA IHC-stained positive areas. Results are the means ± SEM. The Student’s t-test was used to compare two groups. One-way ANOVA was used to compare three or more groups (*P < 0.05, **P < 0.01, ***P < 0.001, *n* = 4)

To understand the attenuating effect of MSCs on murine liver fibrosis, we injected MSCs once a week after CCl_4_ injection for 6 weeks (24 h after CCl_4_ injection). Mice were sacrificed 4 weeks after MSCs injection for analysis. Compared to the CCl_4_ group, mice injected with MSCs exhibited reduced liver scarring, decreased myofibroblast activation (as shown by α-SMA immunohistochemical staining), and lower collagen deposition (evidenced by Masson staining) after 6 weeks (Fig. 3).

### UC-MSC-derived conditional medium (CM^UC-MSC^) suppresses TGF-β-induced *ACTA2*/α-SMA expression in LX-2 cells

**Fig. 4:**
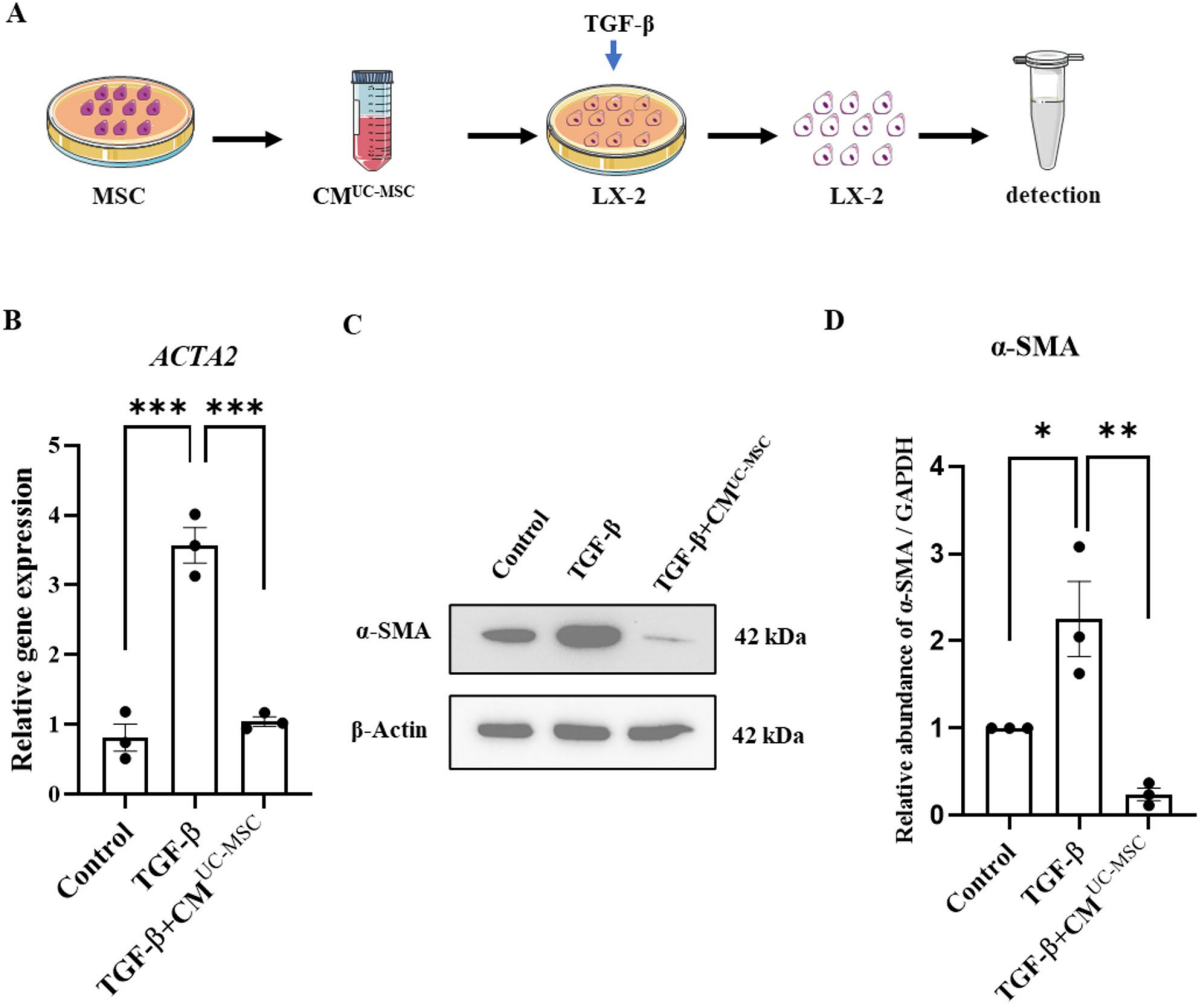
CM^UC-MSC^ suppresses TGF-β-induced ACTA2/a-SMA expression in LX-2 cells. **A** Schematic diagram indicating the time points for the treatment of LX-2 cells. **B** Gene expression of ACTA2 was measured by real-time quantitative PCR. **C**, **D** & Protein expression of α-SMA was measured by Western blotting. Full-length blots/gels are presented in Supplementary Fig.S6. ImageJ was used for quantitative analysis and statistical analysis of western blotting results. Results are the means ± SEM. One-way ANOVA was used to compare three or more groups (*P < 0.05, **P < 0.01, ***P < 0.001, *n* = 3)

As HSCs are the main players in liver fibrosis and CM^UC−MSC^ inhibited liver fibrosis in vivo, we further dissected the influence of CM^UC−MSC^ in vitro. To detect the effect of CM^UC−MSC^ on LX-2, we collected CM^UC−MSC^ and treated LX-2 cells activated by TGF-β (Fig. 4A). Gene expression analysis demonstrated that treatment with CM^UC−MSC^ reduced *ACTA2* (α-SMA) expression, compared to the control group (*p* < 0.0001, Fig. 4). Upon TGF-β induction, *ACTA2* expression increased by 2.8-fold, relative to the control. Compared to the TGF-β-induced group, the expression of *ACTA2* decreased by 85% and 68% at 24 h and 48 h, respectively, after CM^UC−MSC^ treatment (*p* = 0.0002, Fig. 4B and E). Similarly, CM^UC−MSC^-treated LX-2 cells displayed reduced α-SMA protein expression, as confirmed by Western blotting (*p* < 0.05) (*p* < 0.005, Fig. 4C, D, F, G).

### UC-MSC-derived extracellular vesicles (EV^UC-MS^) have limited effects on TGF-β-induced activation of LX-2

**Fig. 5:**
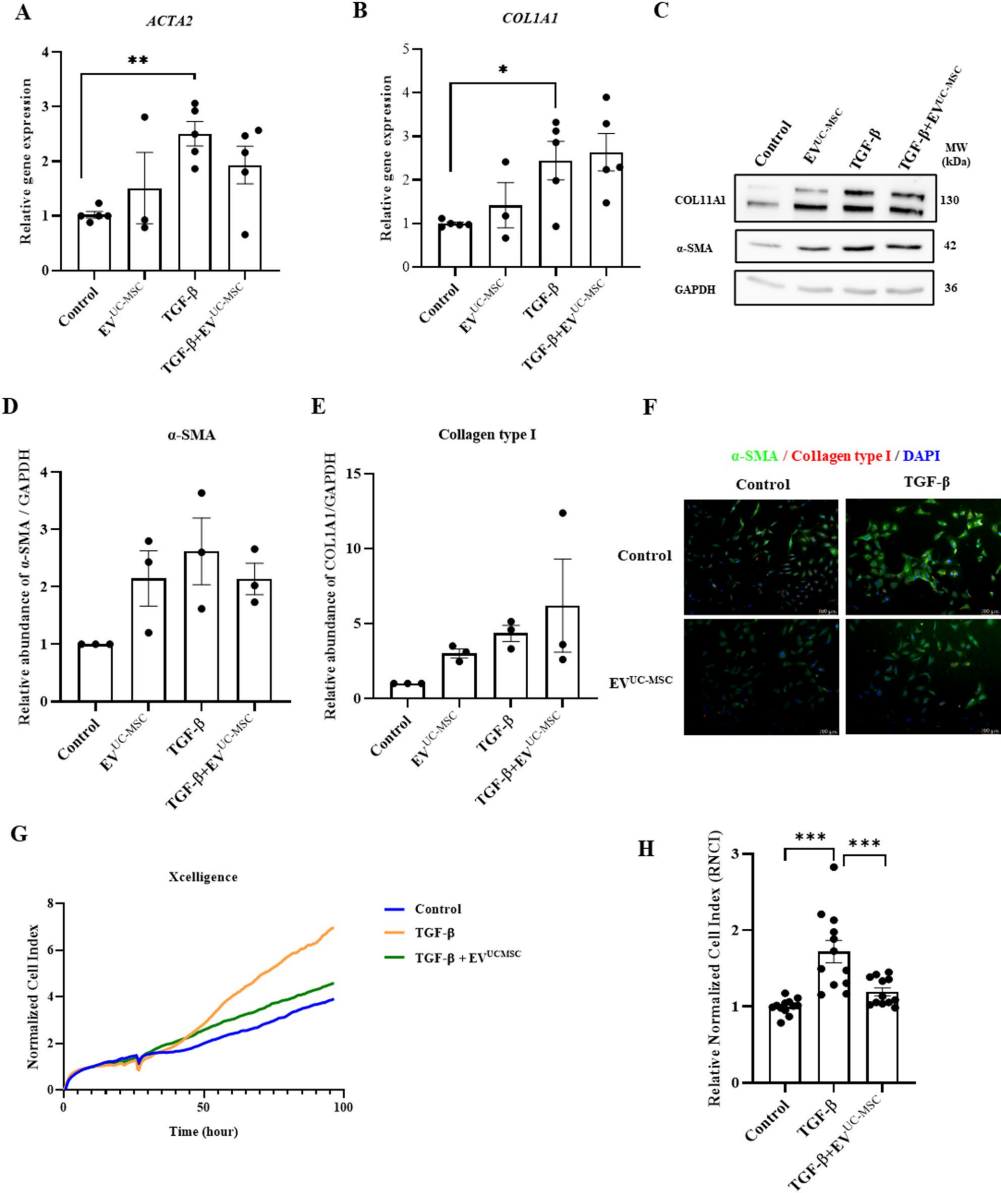
UC-MSC-derived extracellular vesicles (EV^UC-MS^) have limited effects on TGF-β-induced activation of LX-2. LX-2 cells were treated with TGF-β (5 ng/ml) for activation. **A**, **B** ACTA2 and COL1A1 gene expression were measured by real-time quantitative PCR. **C**, **E** α-SMA and COL1A1 were measured by western blotting. Full-length blots/gels are presented in Supplementary Fig.S7. **F** Protein expression of α-SMA and collagen type 1 was determined by immunofluorescence. Scale bar: 100 μm. **G** LX-2 cell proliferation was monitored by a real-time xCELLigence system. **H** The Relative Normalized Cell Index (RNCI) of LX-2 cells in xCELLigence assays. Data are shown as mean ± SEM (*n* = 3). ImageJ was used for quantitative analysis and statistical analysis of Western blot results. Results were the means ± SEM. One-way ANOVA was used to compare differences between three or more groups (*P < 0.05, **P < 0.01, ***P < 0.001. *n* > 3)

Since conditioned medium comprises a ‘soluble’ fraction (SF^UC−MSC^) and a particulate (EV) fraction (EV^UC−MS^), we first explored the effect of EVs on activated LX-2 and controls. EVs were characterized using NTA, which revealed a population size of 197.9 ± 48.6 nm (Fig. S5B&C). NTA and Cryo-EM confirmed the intact structure and similar round morphology of isolated EVs (Fig. S5C&E), while Western blot analysis indicated the presence of EV-specific markers, including CD9 and TSG101 (Fig. S5D). Treatment with EV^UC−MSC^ did not change the expression of *ACTA2* (α-SMA) and *COL1A1* (collagen type I) compared to the control group. Additionally, EV^UC−MSC^ did not change LX-2 activation at either the transcriptional or protein level (Fig. 5A–F). However, a real-time cell proliferation assay showed that EV^UC−MSC^ inhibited TGF-β-induced LX-2 proliferation (Fig. 5G&H).

### UC-MSC-derived soluble factors (SF^UC-MSC^) suppress TGF-β-induced activation of LX-2

**Fig. 6:**
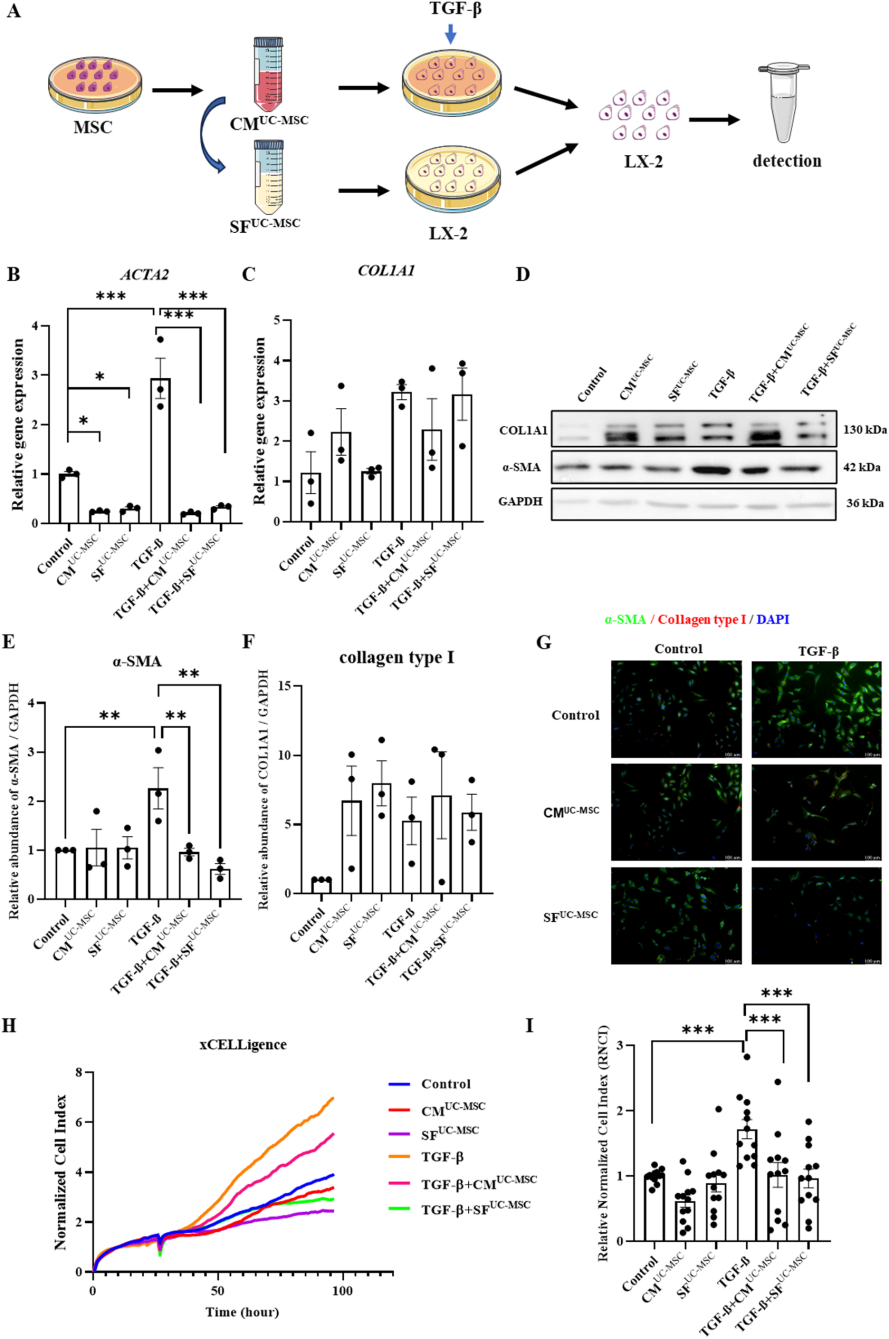
UC-MSC-derived soluble factors (SF^UC-MSC^) suppress TGF-β-induced activation of LX-2. **A** Schematic diagram indicating the time points for the treatment of LX-2 cells. **B** and **C** Gene expression of ACTA2 and COL1A1 was measured by real-time quantitative PCR. **D**–**F** Protein expression of α-SMA was determined by Western blotting. Full-length blots/gels are presented in Supplementary Fig. S8. **G** Protein expression of α-SMA and collagen type 1 was determined by immunofluorescence. Scale bar: 100 μm. **H** LX-2 cell proliferation was monitored by a real-time xCELLigence system. **I** The Relative Normalized Cell Index (RNCI) of LX-2 cells in xCELLigence assays. Data are shown as mean ± SEM (*n* = 3). ImageJ was used for quantitative analysis and statistical analysis of the western blot results. Results are the means ± SED. One-way ANOVA was used to compare three or more groups (*P < 0.05, **P < 0.01, ***P < 0.001. *n* > 3)

Gene expression analysis revealed that treatment with CM^UC−MSC^ and SF^UC−MSC^ suppressed the expression of *ACTA2* (α-SMA), compared to the control group. CM^UC−MSC^ and SF^UC−MSC^ suppressed LX-2 cell activation at both the transcriptional and protein levels. Moreover, compared to the TGF-β-induced group, CM^UC−MSC^ and SF^UC−MSC^ suppressed LX-2 cell activation at both the transcriptional and protein levels (Fig. 6B–E, G). Despite the marked reduction in α-SMA, expression of collagen type I did not change in response to CM^UC−MSC^ and SF^UC−MSC^ treatment (Fig. 6C, D, F, G). Further analysis using real-time cell proliferation assays demonstrated a reduction in the proliferation of CM^UC−MSC^- and SF^UC−MSC^-treated LX-2 cells compared to the TGF-β-induced group (Fig. 6H and I).

## Discussion

In this study, we investigated the preventive and therapeutic effects of UC-MSC injection on liver fibrosis in mice and explored the mechanisms underlying their anti-fibrotic actions in vitro. Our findings demonstrate that UC-MSCs preferentially home to the liver, exert dual effects, i.e. both prevent and attenuate fibrosis, and mediate their anti-fibrotic effects primarily through SF^UC−MSC^ rather than EV^UC−MSC^.

The therapeutic potential of MSCs fundamentally depends on their ability to either physically localize to or functionally influence damaged tissues [10]. Multiple administration routes for MSC transplantation have demonstrated therapeutic efficacy in various liver pathologies. Clinically relevant delivery approaches include: portal vein injection; hepatic artery infusion; intrahepatic injection; intrasplenic administration and intraperitoneal injection [10]. The selection of the delivery route critically influences the engraftment efficiency and subsequent therapeutic potential of MSCs in liver regeneration. Intravenous infusion, while widely used, may limit hepatic homing due to nonspecific cell distribution and pulmonary entrapment. In contrast, intrahepatic, portal vein, or IP administration could enhance targeted engraftment by maximizing MSC retention within the liver microenvironment [29], which we also observed in a direct comparison of these 2 application routes for MSC. Moreover, we observed that applicing high numbers of MSC (>3 million in 300 µl) caused significant lethality in mice, whereas intraperitoneal injection did not. Furthermore, fluorescently labeled MSC tracking revealed that after tail vein injection, MSCs were primarily distributed in the lungs. In contrast, after intraperitoneal injection, MSCs were more predominantly distributed in the liver. Our results suggest that intraperitoneal administration may enhance liver homing, potentially enabling MSCs to exert a more potent local therapeutic effect in liver fibrosis.

Our results showed that after injection, MSCs mainly accumulated in the liver, and more MSCs accumulated in fibrotic livers than in normal livers. This may be due to the homing of MSCs. The mechanism of this homing phenomenon is still unclear. This homing ability is likely mediated by chemokines and adhesion molecules upregulated during liver injury. Chemokines bind to their receptors and are essential for cell adhesion, migration, and assembly of the ECM [30]. Stromal cell-derived factor-1 (SDF-1) is a small chemokine in the CXC chemokine family, which plays a key role in the homing of MSCs [31]. SDF1 can bind to the C-X-C motif chemokine receptor 4 (CXCR4) expressed by MSCs and induce MSCs to mobilize and home to damaged tissues along the concentration gradient of SDF-1, thus playing a therapeutic role [32]. The MSCs injected by IP may be absorbed into the mesenteric microvessels through the peritoneum and enter the liver through the portal vein. The damaged liver releases chemokines such as SDF-1 and HGF, which attract MSCs to home through the CXCR4/SDF-1 axis.

We found that UC-MSCs not only prevent but also attenuate established liver fibrosis in a CCl₄-induced model. This dual effect is supported by histological evidence, including reduced collagen deposition and α-SMA expression. UC-MSCs may act through multiple mechanisms, such as ECM remodeling, immune modulation, and suppression of HSC activation, making them a versatile therapeutic option for liver fibrosis. MSCs secrete a diverse array of bioactive molecules, including cytokines, growth factors, anti-inflammatory factors, and even proteins, which play pivotal roles in mediating the inhibition of liver fibrosis [20]. CM^UC−MSC^ significantly reduces the expression of MMP-2, α-SMA, and type I collagen in primary HSCs, indicating that MSC-derived secretory factors inhibit HSC activation [33]. In mouse models of chronic liver injury, microencapsulated MSC injection reduced inflammation and fibrosis, suggesting that SF^UC−MSC^ mediate these therapeutic effects [34]. Previous studies have demonstrated that MSCs can promote infiltration of host monocytes and neutrophils into the liver, thereby alleviating fibrosis through the synthesis of matrix metalloproteinases (MMPs) [35]. Further evidence indicates that BM-MSC transplantation induces a shift toward M2 macrophage polarization, characterized by MMP13 expression, while suppressing M1 macrophage activity. This dual mechanism inhibits HSC activation and synergistically attenuates carbon tetrachloride (CCl_4_)-induced liver fibrosis [36].

A key finding of our study is the dominance of SF^UC−MSC^ over EV^UC−MSC^ in mediating the anti-fibrotic effects of UC-MSCs. Both CM^UC−MSC^ and SF^UC−MSC^ significantly inhibited TGF-β-induced LX-2 activation and proliferation, while EV^UC−MSC^ had limited effects at low concentrations. Notably, studies have shown the anti-fibrotic properties of MSC-derived EVs in liver fibrosis. These EVs act on different cells and work through a variety of pathways and mechanisms. MSC-derived exosomal miR-27b-3p can attenuate liver fibrosis by downregulating the Yes-associated protein and lysyl oxidase-like 2 (YAP/LOXL2) pathway [37]. MSC-derived exosomes can regulate the macrophage phenotype, modulate the inflammatory microenvironment of the liver, and repair liver injury by delivering miR-148a to block the KLF6/STAT3 pathway in macrophages [18]. MSCs can also inhibit the proliferation and cytokine production of intrahepatic B cells through exosomes, regulate the MAPK and NF-κB signaling pathways, and alleviate liver fibrosis in mice [38]. In our study, TGF-β-activated LX-2 cells exhibited significant inhibition upon treatment with high protein concentrations (10 µg/mL) EV^UC−MSC^ (data not shown). This suggests that the UC-MSC-derived secretome, including EV^UC−MSC^, has a potent inactivating effect on HSCs, and their antifibrotic capacity depends on the composition and dose of these factors. Therefore, EVs secreted by MSCs are highly concentrated before therapeutic application and may not represent the strongest anti-fibrotic potential of the intact MSC secretome. We compared the therapeutic effects of CM^UC−MSC^ (i.e., SF^UC−MSC^+EV^UC−MSC^) with conditioned medium depleted of EVs (i.e., SF^UC−MSC^ alone) and found that EV SF^UC−MSC^ contributed limited to the anti-fibrotic effects of CM^UC−MSC^ (where SF and EVs are at “physiological” levels). Although further in vivo validation is needed, our findings challenge the prevailing focus on EV^UC−MSC^ alone and call for a shift in mechanistic studies to also consider SF^UC−MSC^ as a key therapeutic mediator.

Our study serves as an important step in highlighting the therapeutic potential of soluble factors, which are often discarded as medical waste during EV^UC−MSC^ isolation. We hope this work will prompt further investigations to identify the specific active components within SF^UC−MSC^. In line with previous studies identifying HGF and TSG-6 as anti-fibrotic factors [39–41], we detected high concentrations of HGF (approximately 10 ng/ml) in both CM^UC−MSC^ and SF^UC−MSC^ (Fig. S6), supporting its role as a plausible contributor to the observed effects. MSCs can effectively ameliorate liver damage through potent antioxidant and anti-apoptotic mechanisms, potentially leading to more sustained therapeutic effects [42].

While our study provides valuable insights, several limitations should be acknowledged. First, the specific SF^UC−MSC^ responsible for the observed effects remains to be identified. Proteomic and functional studies are needed to pinpoint the key mediators. Second, the long-term safety and efficacy of SF^UC−MSC^ requires further evaluation in preclinical and clinical settings. In vivo experiments using SF^UC−MSC^ to treat the fibrosis mice are needed to analysis the contribution of SF^UC−MSC^ and are included in our future experimental plans. Finally, the mechanisms underlying MSC homing to the liver and their interactions with resident cells warrant further exploration.

## Conclusions

Administered therapeutic UC-MSCs preferentially accumulate in the liver and are effective in preventing and reversing liver fibrosis in vivo, while inhibiting hepatic stellate cell activation in vitro. In vivo antifibrotic effects may be mediated primarily by excreted soluble factors rather than EVs. These offer broad potential for the future development of cell-free therapies that can be applied in clinical applications.

## Supplementary Information

Supplementary material 1.

## Data Availability

The data supporting the findings of this study are available from the corresponding author upon reasonable request. All data generated or analyzed are included in this article and its supplementary files, including full-length uncropped blots/gels in the supplementary figures.

## Abbreviations

ACTA2: Actin alpha 2
ALT: Alanine aminotransferase
α-SMA: Alpha smooth muscle actin
AST: Aspartate aminotransferase
CCl₄: Carbon tetrachloride
CM: Conditioned medium
COL1A1: Collagen type I alpha 1 chain
CXCR4: C-X-C motif chemokine receptor 4
DiR: 1,1’-Dioctadecyl-3,3,3’,3’-tetramethylindotricarbocyanine iodide
ECM: Extracellular matrix
EV: Extracellular vesicle
H&E: Hematoxylin and eosin
HSC: Hepatic stellate cell
IHC: Immunohistochemical
LOXL2: Lysyl oxidase-like 2
MMPs: Matrix metalloproteinases (MMPs)
MSC: Mesenchymal stem cells
SDF-1: Stromal cell-derived factor-1
SF: Soluble factors
TGF-β: Transforming growth factor beta 1
UC: Umbilical cord
YAP: Yes-associated protein
