# Supplementary material for: Human umbilical cord mesenchymal stem cells attenuate liver fibrosis in mice and inhibit hepatic stellate cell activation by secreting soluble factors
Source: Stem Cell Res Ther. 2025 Nov 27;16:698. doi: 10.1186/s13287-025-04812-6 (PMC12751779; doi:10.1186/s13287-025-04812-6)
Supplement: Supplementary file 1 — Supplementary material 1. [file 13287_2025_4812_MOESM1_ESM.docx]

**Supplementary Figures and Tables**

**Human umbilical cord mesenchymal stem cells attenuate liver fibrosis in mice and inhibit hepatic stellate cell activation by secreting soluble factors**

Qi Zhou ^a, b, d^, Junyu Wang ^a, b^, Ke Cheng ^d^, Ruyi Mei ^d^, Janette Heegsma^a^, Han Moshage ^a^, Martin C. Harmsen ^b, e, f^, Klaas Nico Faber ^a,^ *, and Pingnan Sun ^c,^ ^d^*

a University Medical Center Groningen, University of Groningen, Department of Gastroenterology and Hepatology, Groningen, The Netherlands

b University Medical Center Groningen, University of Groningen, Department of Pathology and Medical Biology, Groningen, The Netherlands

c Department of Gynecology of the First Affiliated Hospital, Shantou University Medical College, Shantou, Guangdong, China

d Department of Stem Cell Research Center, Shantou University Medical College, Shantou, Guangdong, China

e University of Groningen, University Medical Centre Groningen, W.J. Kolff Institute for Biomedical Engineering and Materials Science, Groningen, The Netherlands

f University of Groningen, University Medical Centre Groningen, Groningen Research Institute for Asthma and COPD (GRIAC), Groningen, The Netherlands

***Corresponding author:**

Klaas Nico Faber, k.n.faber@umcg.nl; Pingnan Sun, pnsun@stu.edu.cn

**Supplementary tables**

**Table S1. Sequences of Rat Primers and Probes used for Real-time PCR Analysis**

| **Gene** | **Sense 5’ − 3’** | **Antisense 5’ − 3’** | **Probe 5’ − 3’** |
| --- | --- | --- | --- |
| *ACTA2* | CTGACTGACTACCTCATGAAGATCCT | CTTAATGTCACGCACGATTTCC | CAGCTTCACCACCACGGCCGAG |
| *COL1A1* | GGCCCAGAAGAACTGGTACATC | CCGCCATACTCGAACTGGAA | CCCCAAGGACAAGAGGCATGTCTG |
| *18S* | CGGCTACCACATCCAAGGA | CCAATTACAGGGCCTCGAAA | CGCGCAAATTACCCACTCCCGA |

| **Protein** | **Compony** | **catalog number** |
| --- | --- | --- |
| α-SMA | Sigma Aldrich | A5228 |
| COL1A1 | Southern Biotech | 1310-01 |
| β-Actin | Cell Signaling | 4970 |
| CD9 | Cell Signaling | 13174 |
| TSG101 | Santa Cruz | sc-7964 |
| Calnexin | BD | 610523 |
| GAPDH | Calbiochem | CB1001 |
| α-tubulin | Sigma Aldrich | T9026 |
| Goat Anti- Rabit Immunoglobulins HRP | Dako | P0448 |
| Rabit Anti-Mouse Immunoglobulins HRP | Dako | P0260 |
| Rabit Anti-Goat Immunoglobulins HRP | Dako | P0449 |
| Goat anti-Mouse Secondary Antibody, Alexa Fluor™ Plus 488 | Invitrogen | A-32723 |
| Donkey anti-Goat Secondary Antibody, Alexa Fluor™ Plus 594 | Invitrogen | A-11058 |

**Table S2. The list of antibodies**

**Supplementary figures**

**
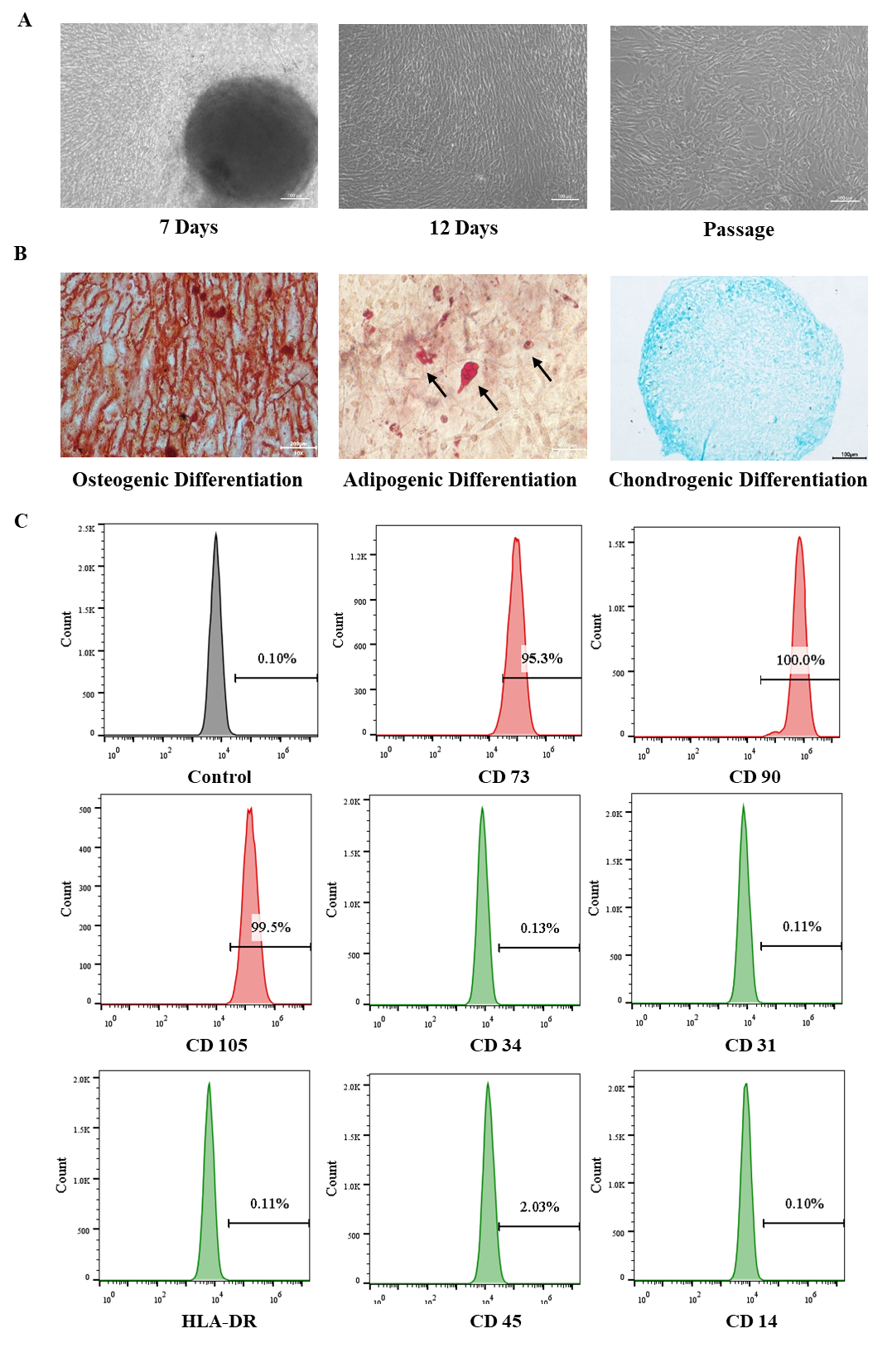
**

**Fig. S1. Characterization of human mesenchymal stem cells.**

(A) Representative images of MSC morphology during MSC isolation. Scale bars,100 µm. (B) Representative images of MSC adipogenic differentiation, osteogenic differentiation and chondrogenic differentiation after MSC differentiation. Differentiated cells were stained with Oil Red O, alizarin red and alician blue. Scale bars,100 µm. (C) Phenotypic analysis of MSC surface markers by flow cytometry staining (red indicates a positive surface marker; green indicates a negative surface marker.


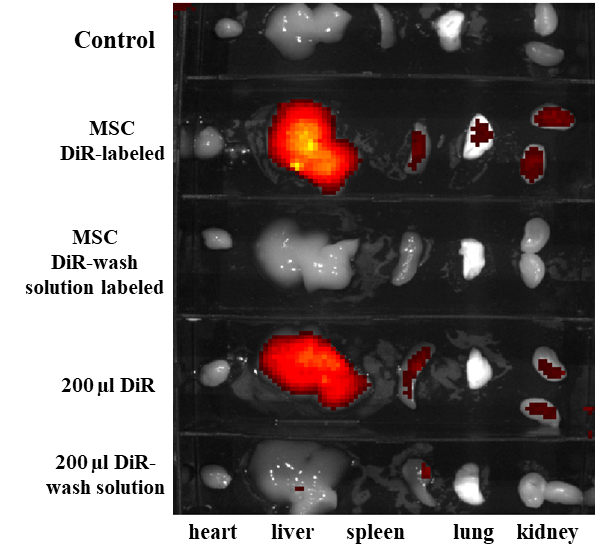


**Fig. S2. The fluorescence signal was detected under the living image after IP injection.**


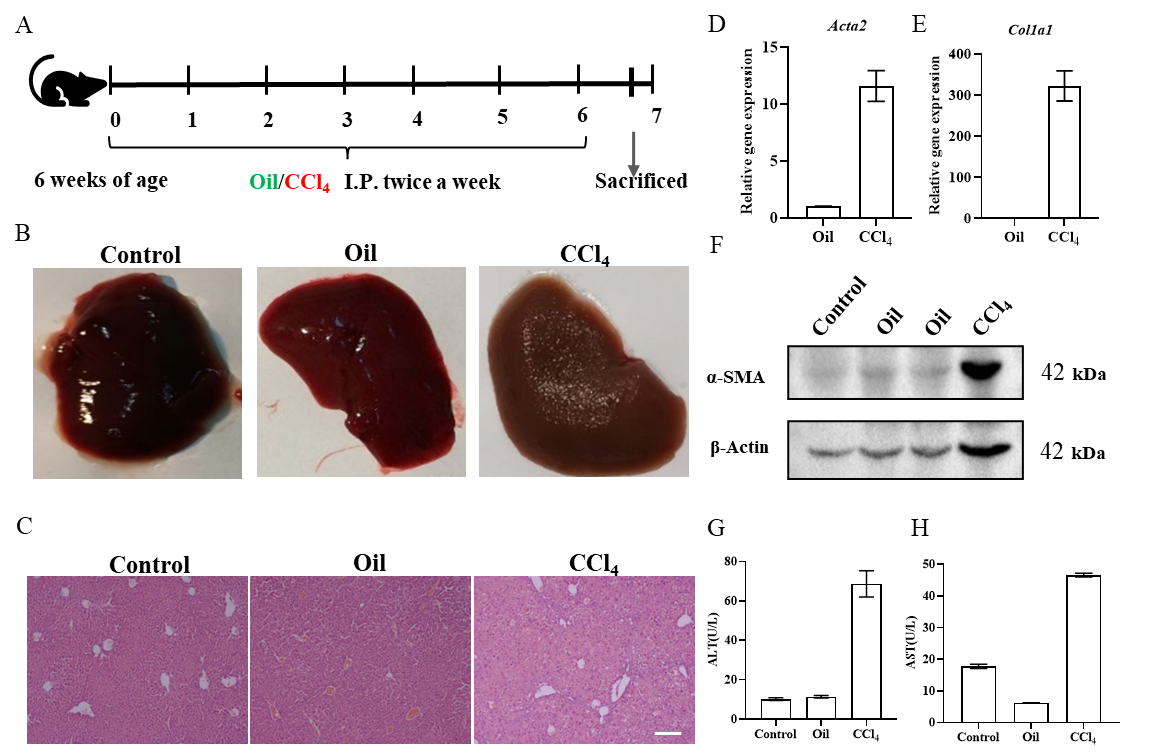


**Fig. S3. Establishment of the CCl_4_-mediated liver fibrosis mouse model.**

(A) Schematic diagram of experimental procedures and time points. (B) Liver morphology after different treatments. (C) Representative images of hematoxylin and eosin-stained liver sections from representative control, oil, CCl_4_ and CCl_4_ + MSC groups. Scale bars: 100 µm. (D&E) qPCR of *ACTA2* and *COL1A1* in the livers from different groups, *GAPDH* was used as house-keeping gene. (F) Western blot of α-SMA and β-actin in the livers from different groups. (G&H) Serum levels of AST and ALT are from different groups.

**Fig. S4. Body weight of the mice during the procedure of the experiment.**


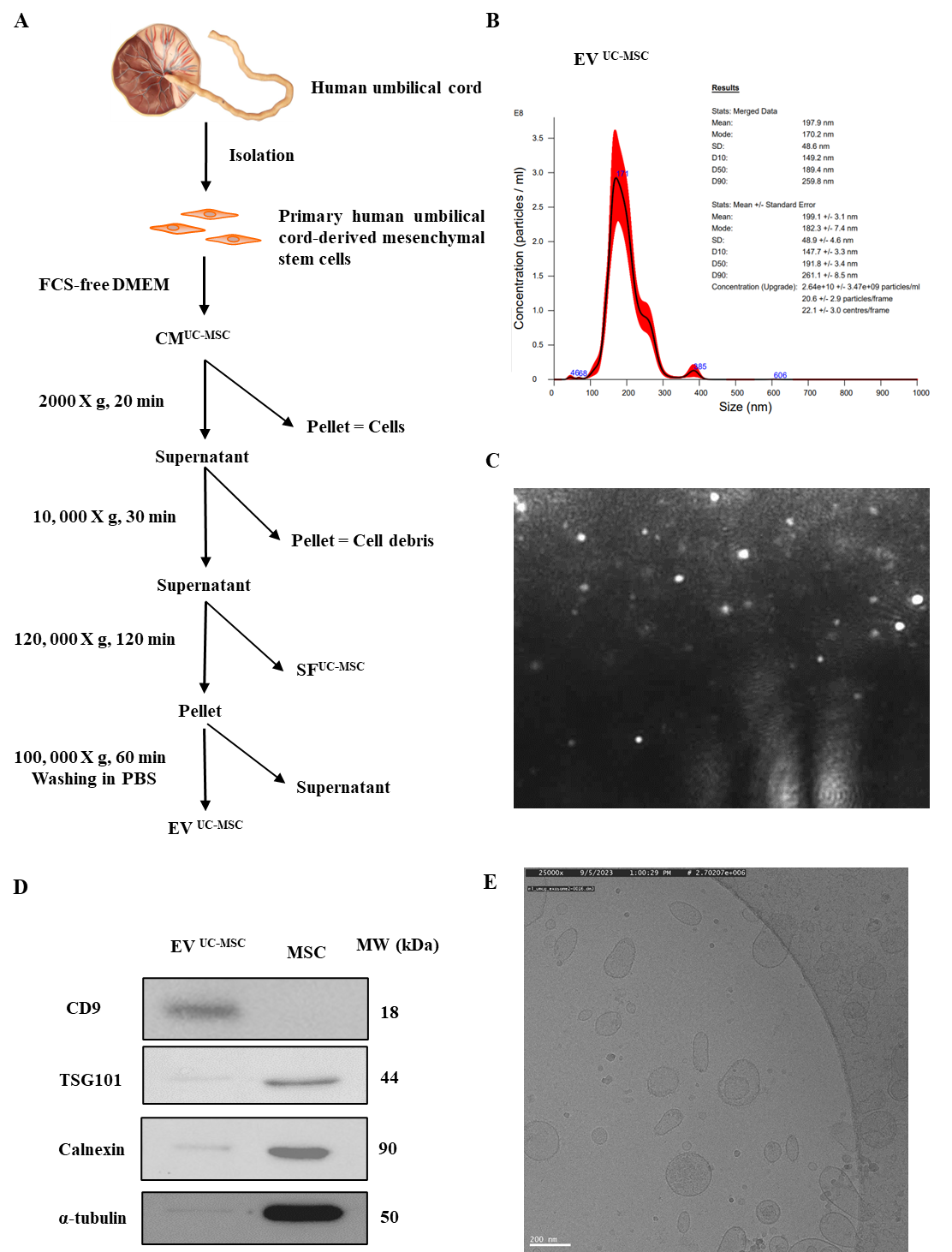


**Fig. S5. Isolation and characterization of MSC-derived extracellular vesicles (EV^UC-MSC^)**

(A) Schematic diagram of extracellular vesicles (EV) extraction experimental steps. (B) Distribution of EV^UC−MSC^ by nanoparticle tracking analysis (NTA). Average diameter of EV^UC−MSC^ was analyzed by NTA (n = 5). (C) Visualization of EV^UC−MSC^ by Nano Tracking analysis. (D) Western blot analysis of EV^UC−MSC^ by CD9, TSG101, calnexin, and α-tubulin. (E) Cryogenic electron microscopy of EV^UC−MSC^ (scale bar = 200 nm).

**Fig. S6. The secretion of HGF were detected by ELISA.**


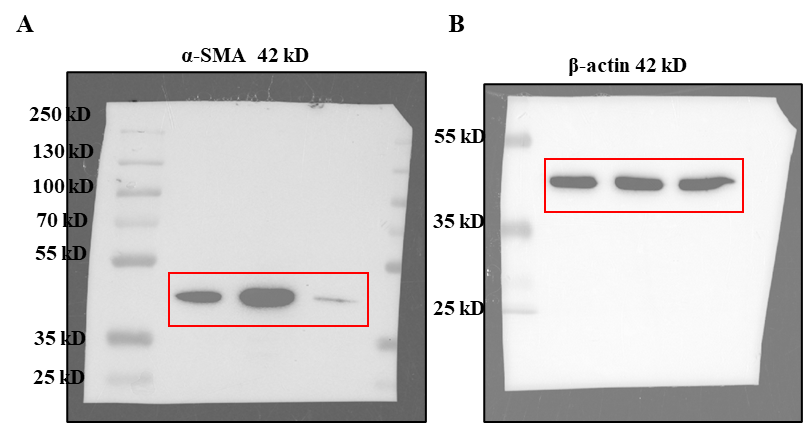


**Fig. S7. Original Western Blot bands in Fig. 4C.** (A) Original Western Blot bands of α-SMA at 42 kD. (B) Original Western Blot bands of β-Actin at 42 kD.


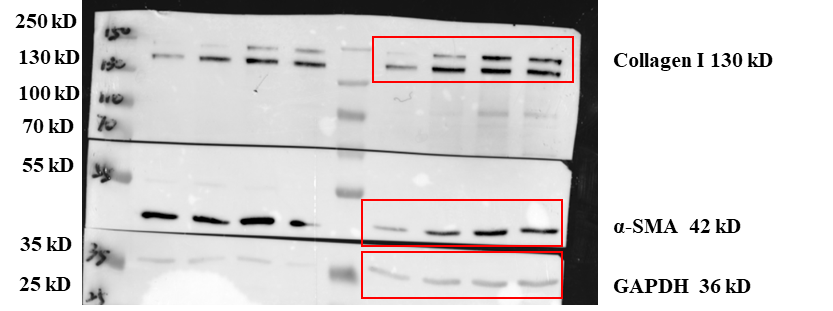


**Fig. S8. Original Western Blot bands in Fig. 5C.** Original Western Blot bands of Collagen I at 130 kD, α-SMA at 42 kD and GAPDH at 36 kD.


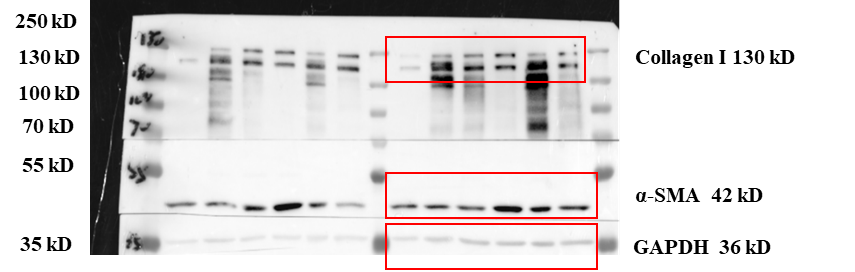


**Fig. S9. Original Western Blot bands in Fig. 5C.** Original Western Blot bands of Collagen I at 130 kD, α-SMA at 42 kD and GAPDH at 36 kD.


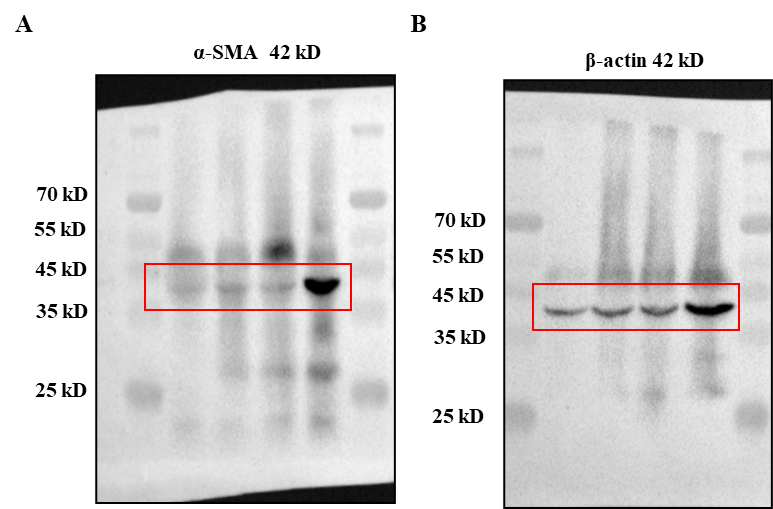


**Fig. S10. Original Western Blot bands in Fig. S3F.** (A) Original Western Blot bands of α-SMA at 42 kD. (B) Original Western Blot bands of β-actin at 42 kD.


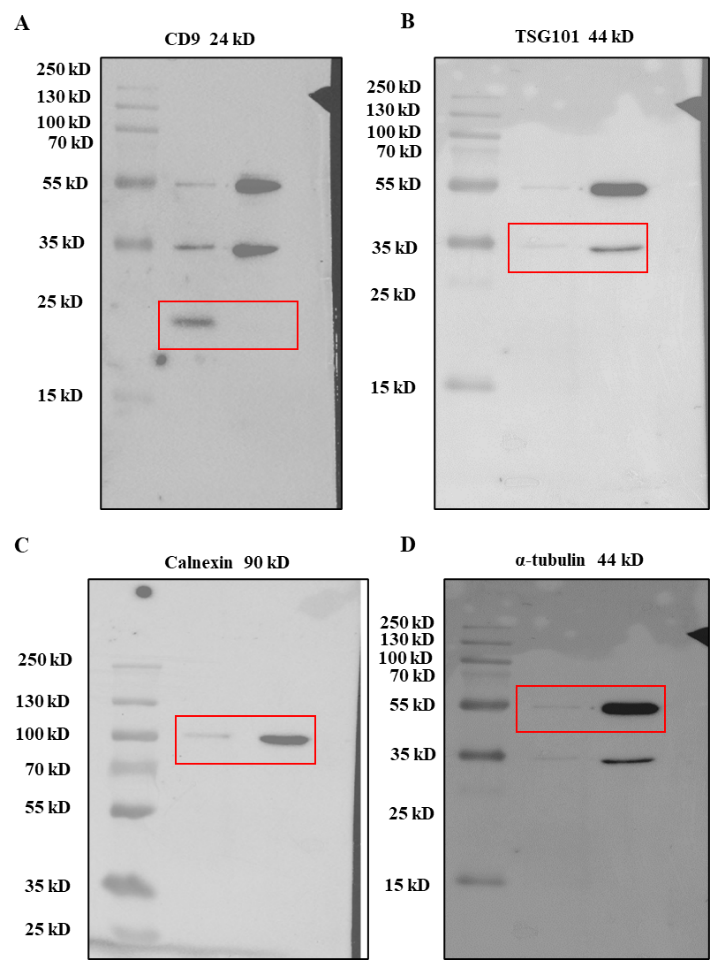


**Fig. S11. Original Western Blot bands in Fig. S5D.** (A) Original Western Blot bands of CD9 at 24 kD. (B) Original Western Blot bands of TSG101 at 44kD. (C) Original Western Blot bands of Calnexin at 90 kD. (D) Original Western Blot bands of α-tubulin at 44 kD.
